# A scientometric analysis of the 100 most cited articles on magnetic resonance guided focused ultrasound

**DOI:** 10.3389/fnhum.2022.981571

**Published:** 2022-09-12

**Authors:** Kanwaljeet Garg, Manish Ranjan, Vibhor Krishna, Manmohan Singh, Ali Rezai

**Affiliations:** ^1^All India Institute of Medical Sciences, New Delhi, India; ^2^Department of Neurosurgery, Rockefeller Neuroscience Institute, West Virginia University, Morgantown, WV, United States; ^3^The University of North Carolina at Chapel Hill, Chapel Hill, NC, United States

**Keywords:** focused ultrasound, neurosurgery, Parkinson’s disease, essential tremor, MRI, FUS

## Abstract

**Background:**

Diagnostic ultrasound has long been a part of a physician’s armamentarium, but transcranial focused ultrasound (FUS) is an emerging treatment of neurological disorders. Consequently, the literature in this field is increasing at a rapid pace.

**Objective:**

This analysis was aimed to identify the top-cited articles on FUS to discern their origin, spread, current trends highlighting future impact of this novel neurosurgical intervention.

**Methods:**

We searched the *Web of Science* database on 28th May 2021 and identified the top 100 cited articles. These articles were analyzed with various scientometric parameters like the authors, corresponding authors, country of corresponding author, journal of publication, year of publication. Citation based parameters including total citations, mean citations per article and mean citations, citation count, and the citation per year, citations per year and co-authors per document were studied as well in addition to Hirsch h-index, g-index, m-index, Bradford’s Law, Lotka’s law and Collaboration index.

**Results:**

The 100 top-cited articles were published between 1998 and 2019 in 45 different journals. The average citations per document and citations per document per year were 97.78 and 12.47, respectively. The most prolific authors were Hynynen K (Medical Biophysics—Toronto), Elias WJ (Neurosurgery—Virginia), Zadicario (InSightec). The Journal of Neurosurgery published the most top-cited articles (*n* = 11), and most articles originated from the United States, followed by Canada. Among individual institutions, the University of Toronto was the most productive.

**Conclusion:**

FUS is an emerging treatment of neurological disorders. With its increasing application, the FUS literature is increasing rapidly. Eleven countries contributed to the top 100 cited articles, with the top 2 countries (the United States and Canada) contributing to more than half of these articles.

## Introduction

Diagnostic ultrasound has been a part of a physician’s armamentarium for the last seven decades. Focsused ultrasound (FUS) has emerged as a therapeutic option in the last few decades to successfully ablate soft tissue tumors such as uterine fibroids, breast carcinoma, and bone metastases (Fry et al., 1958; Fry and Fry, 1960; Cline et al., 1992, 1993, 1995; Hynynen et al., 1993a,b). Technological advances including development of a phased spherical array with a multielement transducer helmet and the implementation of magnetic resonance imaging guidance for real-time tissue temperature monitoring led to intracranial application for neurological disorders, specifically the movement disorders (Clement and Hynynen, 2002; Lipsman et al., 2014). FUS has evolved rapidly in the recent years, specifically in the functional neurosurgery with significant clinical and research publication.

Bibliometrics is a methodological approach from the library sciences that statistically analyses the citation counts of books, articles, and other publications to determine the influence and impact of the scientific publications. Scientometrics is a subfield of bibliometrics that studies science publications by using bibliometric methods to find author, article, and journal-level metrics (i.e., H-index, citation index, and journal impact factor, respectively). It provides a broad overview of the field’s direction, complements the expert peer review process, and is transparent and objective. Many scientometric analyses have been published in neurosurgery to find the 100-most cited articles on topics like endoscopic third ventriculostomy, ossified posterior longitudinal ligament, pallidotomy, and cervical spondylotic myelopathy (Zagzoog et al., 2018; Chen et al., 2019; Agrawal et al., 2021; Garg et al., 2021, 2022a,b; Zhao et al., 2021). However, there is no such article published on transcranial FUS. This scientometric analysis presents the top 100 cited articles published on transcranial FUS and further reports the most significant contributors (authors, institutes, and countries) along with the various scientometric indices.

## Materials and methods

### Search strategy

A systematic search of the *Web of Science* database was performed on 28th May 2021. The keywords used for literature search were—“MR guided focused ultrasound,” “MRgFUS,” “Magnetic resonance-guided focused ultrasound,” “Ultrasound Thalamotomy,” “Ultrasound Thalamotomy for Essential Tremor,” “Ultrasound Subthalamotomy, “focused ultrasound tremor,” “focused ultrasound tremor,” “ultrasound Parkinson,” “focused ultrasound ablation,” “transcranial focused ultrasound,” “focused ultrasound neuromodulation” and “focused ultrasound blood brain barrier opening.” The search results were screened and arranged in descending order of the number of citations, and articles were selected as per the following inclusion and exclusion criteria. FUS studies on transcranial FUS for neurological disorders, comparative study of FUS with DBS or radiofrequency (RF) for movement disorders, blood-brain barrier permeability, animal or cadaver research, targeted therapeutics and radiological aspect of FUS were included. FUS articles on non-cranial pathology were excluded.

### Data and bibliometric parameters studied

The articles were arranged in descending order according to the number of citations. The various parameters analyzed were the title of the articles, authors, corresponding authors, country of corresponding author, journal of publication, year of publication. Citation based parameters including total citations, mean citations per article and mean citations, citation count, and the citation per year, citations per year and co-authors per document were studied as well. The following statistical parameters were considered during the analysis:

Hirsch h-index: authors number of publications and number of citations, reviewed in other articles.

g-index: is a variant of h-index which gives credit for the most cited papers. It is the highest rank where the sum of the citations is larger than the square of rank.

m-index: is another variant of the h-index that displays h-index per year since first publication.

Bradford’s Law: estimates the exponentially diminishing returns of searching for references in science journals was also studied.

Lotka’s law: which denotes the distribution of the number of articles published by the number of authors.

Collaboration index: it assigns a weighted credit to each author in a multi-author paper to capture a researcher’s scientific caliber better.

### Analysis

The statistical analysis was performed using R software version 4.0.3 (R Foundation for Statistical Computing, Vienna, Austria) (Aria and Cuccurullo, 2017; R Core Team, 2022).

## Results

### Articles

The literature search yielded 2,500 articles, and we selected the 100 most cited articles which met the study inclusion and exclusion criteria (Hynynen et al., 2001, 2006; McDannold et al., 2005, 2010; Jordão et al., 2010; Bystritsky et al., 2011; Yoo et al., 2011; Jeanmonod et al., 2012; Park et al., 2012; Treat et al., 2012; Fan et al., 2013; Nance et al., 2014; Elias et al., 2016; Kovacs et al., 2017; Mainprize et al., 2019; Alzheimer’s disease in a mouse model: MR imaging-guided focused ultrasound targeted to the hippocampus opens the blood-brain barrier and improves pathologic abnormalities and behavior—PubMed). The included top-cited articles are summarized in Table 1.

**TABLE 1:** Summary of the included articles.

Paper	DOI	Article type	Journal	Publication year	Corresponding author	University of corresponding author	Total citations	TC per year	Normalized TC	Type of study	Theme of study	Title of article
HYNYNEN K, 2001, RADIOLOGY	10.1148/radiol.2202001804	ARTICLE	RADIOLOGY	2001	HYNYNEN, K	HARVARD UNIV, USA, USA	855	40.71	1	Animal	Blood-brain barrier	Noninvasive MR imaging–guided focal opening of the blood-brain barrier in rabbits

ELIAS WJ, 2013, NEW ENGL J MED	10.1056/NEJMoa1300962	ARTICLE	NEW ENGL J MED	2013	ELIAS, WJ	UNIV VIRGINIA,	385	42.78	2.984	Human	Movement disorder	A pilot study of focused ultrasound thalamotomy for essential tremor
ELIAS WJ, 2016, NEW ENGL J MED	10.1056/NEJMoa1600159	ARTICLE	NEW ENGL J MED	2016	ELIAS, WJ	UNIV VIRGINIA,	376	62.67	3.547	Human	Movement disorder	A randomized trial of focused ultrasound thalamotomy for essential tremor
MCDANNOLD N, 2010, NEUROSURGERY	10.1227/01.NEU.0000360379.95800.2F	ARTICLE	NEUROSURGERY	2010	MCDANNOLD, N	HARVARD UNIV, USA	360	30	2.059	Human	Oncology	Transcranial magnetic resonance imaging– guided focused ultrasound surgery of brain tumors: initial findings in 3 patients
LIPSMAN N, 2013, LANCET NEUROL	10.1016/S1474-4422(13)70048-6	ARTICLE	LANCET NEUROL	2013	LOZANO, AM	UNIV TORONTO, CANADA	310	34.44	2.403	Human	Movement disorder	MR-guided focused ultrasound thalamotomy for essential tremor: a proof-of-concept study
MARTIN E, 2009, ANN NEUROL	10.1002/ana.21801	ARTICLE	ANN NEUROL	2009	MARTIN, E	UNIV CHILDRENS HOSP ZURICH,	286	22	2.424	Human	Pain	High-intensity focused ultrasound for noninvasive functional neurosurgery
LIU HL, 2010, P NATL ACAD SCI USA	10.1073/pnas.1003388107	ARTICLE	P NATL ACAD SCI USA	2010	CHEN, PY	CHANG GUNG UNIV, COLL MED	282	23.5	1.613	Review - Animal	Blood-brain barrier	Magnetic resonance monitoring of focused ultrasound/magnetic nanoparticle targeting delivery of therapeutic agents to the brain
YOO SS, 2011, NEUROIMAGE	10.1016/j.neuroimage.2011.02.058	ARTICLE	NEUROIMAGE	2011	YOO, SS	HARVARD UNIV, USA	265	24.09	1.205	Animal	Stimulation	Focused ultrasound modulates region-specific brain activity
HYNYNEN K, 2006, J NEUROSURG	10.3171/jns.2006.105.3.445	ARTICLE	J NEUROSURG	2006	HYNYNEN, K	UNIV TORONTO, CANADA	231	14.44	1.351	Animal	Blood-brain barrier	Focal disruption of the blood–brain barrier due to 260-kHz ultrasound bursts: a method for molecular imaging and targeted drug delivery
MCDANNOLD N, 2005, ULTRASOUND MED BIOL	10.1016/j.ultrasmedbio.2005.07.010	ARTICLE	ULTRASOUND MED BIOL	2005	MCDANNOLD, N	HARVARD UNIV, USA, USA	225	13.24	1	Animal	Blood-brain barrier	MRI-guided targeted blood-brain barrier disruption with focused ultrasound: Histological findings in rabbits
JORDAO JF, 2010, PLOS ONE	10.1371/journal.pone.0010549	ARTICLE	PLOS ONE	2010	JORDAO, JF	SUNNYBROOK RES INST, TORONTO	221	18.42	1.264	Animal	Alzheimer’s disease	Antibodies targeted to the brain with image-guided focused ultrasound reduces amyloid-beta plaque load in the TgCRND8 mouse model of Alzheimer’s disease
LIPSMAN N, 2018, NAT COMMUN	10.1038/s41467-018-04529-6	ARTICLE	NAT COMMUN	2018	SUNNYBROOK RES INST	UNIV TORONTO, CANADA	213	53.25	3.252	Human	Alzheimer’s disease	Blood–brain barrier opening in Alzheimer’s disease using MR-guided focused ultrasound
JEANMONOD D, 2012, NEUROSURG FOCUS	10.3171/2011.10.FOCUS11248	ARTICLE	NEUROSURG FOCUS	2012	JEANMONOD, D	CTR ULTRASOUND FUNCT NEUROSURG, SWITZERLAND.	195	19.5	2.27	Human	Pain	Transcranial magnetic resonance imaging–guided focused ultrasound: noninvasive central lateral thalamotomy for chronic neuropathic pain
BYSTRITSKY A, 2011, BRAIN STIMUL	10.1016/j.brs.2011.03.007	REVIEW	BRAIN STIMUL	2011	BYSTRITSKY, A	UNIV CALIF LOS ANGELES, LOS ANGELES, USA	175	15.91	0.795	Human	Neurostimulation	A review of low-intensity focused ultrasound pulsation
FAN CH, 2013, BIOMATERIALS	10.1016/j.biomaterials.2013.01.099	ARTICLE	BIOMATERIALS	2013	YEH, CK	NATL TSING HUA UNIV, TAIWAN	158	17.56	1.225	Animal	Blood-brain barrier	SPIO-conjugated, doxorubicin-loaded microbubbles for concurrent MRI and focused-ultrasound enhanced brain-tumor drug delivery
TREAT LH, 2012, ULTRASOUND MED BIOL	10.1016/j.ultrasmedbio.2012.04.015	ARTICLE	ULTRASOUND MED BIOL	2012	MCDANNOLD, N	HARVARD UNIV, USA, USA	158	15.8	1.84	Animal	Blood-brain barrier	Improved anti-tumor effect of liposomal doxorubicin after targeted blood-brain barrier disruption by mri-guided focused ultrasound in rat glioma
HYNYNEN K, 2006, EUR J RADIOL	10.1016/j.ejrad.2006.04.007	ARTICLE	EUR J RADIOL	2006	HYNYNEN, K	HARVARD UNIV, USA, USA	157	9.81	0.918	Animal	Technique	Pre-clinical testing of a phased array ultrasound system for MRI-guided noninvasive surgery of the brain–a primate study
KOVACS ZI, 2017, P NATL ACAD SCI USA	10.1073/pnas.1614777114	ARTICLE	P NATL ACAD SCI USA	2017	KOVACS, ZI	NIH, FRANK LAB, RADIOL & IMAGING SCI, US	143	28.6	2.566	Animal	Blood-brain barrier	Disrupting the blood–brain barrier by focused ultrasound induces sterile inflammation
NANCE E, 2014, J CONTROL RELEASE	10.1016/j.jconrel.2014.06.031	ARTICLE	J CONTROL RELEASE	2014	PRICE, RJ	UNIV VIRGINIA, USA	131	16.38	2.012	Animal	Blood-brain barrier	Non-invasive delivery of stealth, brain-penetrating nanoparticles across the blood-brain barrier using MRI-guided focused ultrasound
BURGESS A, 2014, RADIOLOGY	10.1148/radiol.14140245	ARTICLE	RADIOLOGY	2014	BURGESS, A	SUNNYBROOK RES INST, TORONTO	130	16.25	1.997	Animal	Alzheimer’s disease	Alzheimer’s disease in a mouse model: MR imaging–guided focused ultrasound targeted to the hippocampus opens the blood-brain barrier and improves pathologic abnormalities and behavior
MAINPRIZE T, 2019, SCI REP-UK	10.1038/s41598-018-36340-0	ARTICLE	SCI REP-UK	2019	MAINPRIZE, T	SUNNYBROOK HLTH SCI CTR, CANADA	129	43	2.449	Human	Oncology	Blood-brain barrier opening in primary brain tumors with non-invasive MR-guided focused ultrasound: a clinical safety and feasibility study
RAM Z, 2006, NEUROSURGERY	10.1227/01.NEU.0000254439.02736.D8	ARTICLE	NEUROSURGERY	2006	RAM, Z	TEL AVIV SOURASKY MED CTR, ISRAEL	125	7.81	0.731	Human	Oncology	Magnetic resonance imaging-guided, high-intensity focused ultrasound for brain tumor therapy
PARK EJ, 2012, J CONTROL RELEASE	10.1016/j.jconrel.2012.09.007	ARTICLE	J CONTROL RELEASE	2012	PARK, EJ	HARVARD UNIV, USA, USA	113	11.3	1.316	Animal	Blood-brain barrier	Ultrasound-mediated blood-brain/blood-tumor barrier disruption improves outcomes with trastuzumab in a breast cancer brain metastasis model
LEINENGA G, 2016, NAT REV NEUROL	10.1038/nrneurol.2016.13	REVIEW	NAT REV NEUROL	2016	GOTZ, J	UNIV QUEENSLAND, AUSTRALIA	109	18.17	1.028	Review		Ultrasound treatment of neurological diseases — current and emerging applications
CHANG WS, 2015, J NEUROL NEUROSUR PS	10.1136/jnnp-2014-307642	ARTICLE	J NEUROL NEUROSUR PS	2015	CHANG, JW	YONSEI UNIV, SOUTH KOREA	109	15.57	1.697	Human	Movement disorder	Unilateral magnetic resonance guided focused ultrasound thalamotomy for essential tremor: practices and clinicoradiological outcomes
OBESO JA, 2017, MOVEMENT DISORD	10.1002/mds.27115	REVIEW	MOVEMENT DISORD	2017	OBESO, JA	HOSP UNIV HM PUERTA SUR, SPAIN	102	20.4	1.83	Review	Movement disorder	Past, present, and future of Parkinson’s disease: a special essay on the 200th anniversary of the shaking palsy
MCDANNOLD N, 2007, ULTRASOUND MED BIOL	10.1016/j.ultrasmedbio.2006.10.004	ARTICLE	ULTRASOUND MED BIOL	2007	MCDANNOLD, N	BRIGHAM & WOMENS HOSP, USA	96	6.4	1.401	Animal	Blood-brain barrier	Use of ultrasound pulses combined with definity for targeted blood-brain barrier disruption: a feasibility study
JUNG HH, 2015, MOL PSYCHIATR	10.1038/mp.2014.154	ARTICLE	MOL PSYCHIATR	2015	KIM, CH	INST BEHAV SCI MED, SOUTH KOREA	88	12.57	1.37	Human	OCD	Bilateral thermal capsulotomy with MR-guided focused ultrasound for patients with treatment-refractory obsessive-compulsive disorder: a proof-of-concept study
BOND AE, 2017, JAMA NEUROL	10.1001/jamaneurol.2017.3098	ARTICLE	JAMA NEUROL	2017	ELIAS, WJ	UNIV VIRGINIA, USA	87	17.4	1.561	Human	Movement disorder	Safety and efficacy of focused ultrasound thalamotomy for patients with medication-refractory, tremor-dominant Parkinson’s disease
SUN T, 2017, P NATL ACAD SCI USA	10.1073/pnas.1713328114	ARTICLE	P NATL ACAD SCI USA	2017	SUN, T	HARVARD UNIV, USA, USA	87	17.4	1.561	Animal	Blood-brain barrier	Closed-loop control of targeted ultrasound drug delivery across the blood–brain/tumor barriers in a rat glioma model
MEAD BP, 2016, J CONTROL RELEASE	10.1016/j.jconrel.2015.12.034	ARTICLE	J CONTROL RELEASE	2016	PRICE, RJ	UNIV VIRGINIA, USA	81	13.5	0.764	Animal	Blood-brain barrier	Targeted gene transfer to the brain via the delivery of brain-penetrating DNA nanoparticles with focused ultrasound
ZAAROOR M, 2018, J NEUROSURG	10.3171/2016.10.JNS16758	ARTICLE	J NEUROSURG	2018	ZAAROOR, M	RAMBAM HLTH CARE CAMPUS, ISRAEL	80	20	1.221	Human	Movement disorder	Magnetic resonance-guided focused ultrasound thalamotomy for tremor: a report of 30 Parkinson’s disease and essential tremor cases
MARSAC L, 2012, MED PHYS	10.1118/1.3678988	ARTICLE	MED PHYS	2012	MARSAC, L	UNIV PARIS, FRANCE	79	7.9	0.92	Human	Technique	MR-guided adaptive focusing of therapeutic ultrasound beams in the human head
MEI J, 2009, J ULTRAS MED	10.7863/jum.2009.28.7.871	ARTICLE	J ULTRAS MED	2009	CHENG, Y	CHONGQING MED UNIV, PEOPLES R CHINA	76	5.85	0.644	Animal	Blood-brain barrier	Experimental study on targeted methotrexate delivery to the rabbit brain via magnetic resonance imaging–guided focused ultrasound
JAGANNATHAN J, 2009, NEUROSURGERY	10.1227/01.NEU.0000336766.18197.8E	REVIEW	NEUROSURGERY	2009	KASSELL, NF	UNIV VIRGINIA, USA	76	5.85	0.644	Review		High-intensity focused ultrasound surgery of the brain: part 1—a historical perspective with modern applications
CHANG WS, 2016, J NEUROSURG	10.3171/2015.3.JNS142592	ARTICLE	J NEUROSURG	2016	CHANG, JW	YONSEI UNIV, SOUTH KOREA	75	12.5	0.708	Human	Technique	Factors associated with successful magnetic resonance-guided focused ultrasound treatment: efficiency of acoustic energy delivery through the skull
ARVANITIS CD, 2013, PHYS MED BIOL	10.1088/0031-9155/58/14/4749	ARTICLE	PHYS MED BIOL	2013	ARVANITIS, CD	HARVARD UNIV, USA, USA	75	8.33	0.581	Human	Blood-brain barrier	Combined ultrasound and MR imaging to guide focused ultrasound therapies in the brain
MARTINEZ-FERNANDEZ R, 2018, LANCET NEUROL	10.1016/S1474-4422(17)30403-9	ARTICLE	LANCET NEUROL	2018	OBESO, JA	UNIV HOSP HM PUERTA DEL SUR, SPAIN	72	18	1.099	Human	Movement disorder	Focused ultrasound subthalamotomy in patients with asymmetric Parkinson’s disease: a pilot study
GHANOUNI P, 2015, AM J ROENTGENOL	10.2214/AJR.14.13632	REVIEW	AM J ROENTGENOL	2015	WINTERMARK, M	STANFORD UNIV, USA	71	10.14	1.106	Review		Transcranial MRI-guided focused ultrasound: a review of the technologic and neurologic applications
SCARCELLI T, 2014, BRAIN STIMUL	10.1016/j.brs.2013.12.012	ARTICLE	BRAIN STIMUL	2014	HYNYNEN, K	UNIV TORONTO, CANADA	70	8.75	1.075	Animal	Alzheimer’s disease	Stimulation of hippocampal neurogenesis by transcranial focused ultrasound and microbubbles in adult mice
KONOFAGOU EE, 2012, CURR PHARM BIOTECHNO	NA	REVIEW	CURR PHARM BIOTECHNO	2012	KONOFAGOU, EE	COLUMBIA UNIV, USA	70	7	0.815	Review	Blood-brain barrier	Ultrasound-induced blood-brain barrier opening
ABRAHAO A, 2019, NAT COMMUN	10.1038/s41467-019-12426-9	ARTICLE	NAT COMMUN	2019	ABRAHAO, A	UNIV TORONTO, CANADA	69	23	1.31	Human	Blood-brain barrier	First-in-human trial of blood–brain barrier opening in amyotrophic lateral sclerosis using MR-guided focused ultrasound
MONTEITH S, 2013, J NEUROSURG	10.3171/2012.10.JNS12449	REVIEW	J NEUROSURG	2013	MONTEITH, S	UNIV VIRGINIA, USA	67	7.44	0.519	Review		Potential intracranial applications of magnetic resonance–guided focused ultrasound surgery
HERTZBERG Y, 2010, MED PHYS	10.1118/1.3395553	ARTICLE	MED PHYS	2010	NAVON, G	TEL AVIV UNIV, ISRAEL	67	5.58	0.383	Animal	Technique	Ultrasound focusing using magnetic resonance acoustic radiation force imaging: application to ultrasound transcranial therapy
LARRAT B, 2010, PHYS MED BIOL	10.1088/0031-9155/55/2/003	ARTICLE	PHYS MED BIOL	2010	LARRAT, B	UNIV PARIS, FRANCE	65	5.42	0.372	Animal	Technique	MR-guided transcranial brain HIFU in small animal models
FAN CH, 2016, THERANOSTICS	10.7150/thno.15297	ARTICLE	THERANOSTICS	2016	YEH, CK	NATL TSING HUA UNIV, TAIWAN	64	10.67	0.604	Animal	Blood-brain barrier	Ultrasound/magnetic targeting with SPIO-DOX-microbubble complex for image-guided drug delivery in brain tumors
KRISHNA V, 2018, JAMA NEUROL	10.1001/jamaneurol.2017.3129	REVIEW	JAMA NEUROL	2018	KRISHNA, V	OHIO STATE UNIV, USA	63	15.75	0.962	Review		A review of the current therapies, challenges, and future directions of transcranial focused ultrasound technology
WINTERMARK M, 2014, AM J NEURORADIOL	10.3174/ajnr.A3808	ARTICLE	AM J NEURORADIOL	2014	WINTERMARK, M	UNIV VIRGINIA, USA	63	7.88	0.968	Human	Movement disorder	Imaging findings in MR imaging–guided focused ultrasound treatment for patients with essential tremor
FAN CH, 2016, SCI REP-UK	10.1038/srep19579	ARTICLE	SCI REP-UK	2016	LIU HL	CHANG GUNG UNIV, TAIWAN	62	10.33	0.585	Animal	Blood-brain barrier	Noninvasive, targeted and non-viral ultrasound-mediated GDNF-plasmid delivery for treatment of Parkinson’s disease
HUSS DS, 2015, MOVEMENT DISORD	10.1002/mds.26455	ARTICLE	MOVEMENT DISORD	2015	ELIAS, WJ	UNIV VIRGINIA, USA	60	8.57	0.934	Human	Movement disorder	Functional assessment and quality of life in essential tremor with bilateral or unilateral DBS and focused ultrasound thalamotomy
HUANG Q, 2012, EXP NEUROL	10.1016/j.expneurol.2011.10.027	ARTICLE	EXP NEUROL	2012	CHENG, Y	CHONGQING MED UNIV, PEOPLES R CHINA	58	5.8	0.675	Animal	Blood-brain barrier	Targeted gene delivery to the mouse brain by MRI-guided focused ultrasound-induced blood–brain barrier disruption
SAMIOTAKI G, 2015, J CEREBR BLOOD F MET	10.1038/jcbfm.2014.236	ARTICLE	J CEREBR BLOOD F MET	2015	KONOFAGOU, EE	COLUMBIA UNIV, USA	57	8.14	0.888	Animal	Blood-brain barrier	Enhanced delivery and bioactivity of the neurturin neurotrophic factor through focused ultrasound—mediated blood—brain barrier opening *in vivo*
CHANG JW, 2018, ANN NEUROL	10.1002/ana.25126	ARTICLE	ANN NEUROL	2018	CHANG, JW	YONSEI UNIV, SOUTH KOREA	55	13.75	0.84	Human	Movement disorder	A prospective trial of magnetic resonance–guided focused ultrasound thalamotomy for essential tremor: results at the 2-year follow-up
JONES RM, 2018, THERANOSTICS	10.7150/thno.24911	ARTICLE	THERANOSTICS	2018	JONES, RM	SUNNYBROOK RES INST, CANADA	55	13.75	0.84	Animal	Blood-brain barrier	Three-dimensional transcranial microbubble imaging for guiding volumetric ultrasound-mediated blood-brain barrier opening
KYRIAKOU A, 2014, INT J HYPERTHER	10.3109/02656736.2013.861519	REVIEW	INT J HYPERTHER	2014	KYRIAKOU, A	ITIS FDN RES INFORMAT TECHNOL SOC, SWITZERLAND	55	6.88	0.845	Review		A review of numerical and experimental compensation techniques for skull-induced phase aberrations in transcranial focused ultrasound
XIE F, 2008, ULTRASOUND MED BIOL	10.1016/j.ultrasmedbio.2008.05.004	ARTICLE	ULTRASOUND MED BIOL	2008	PORTER, TR	UNIV NEBRASKA, USA	55	3.93	1	Animal	Blood-brain barrier	Effects of transcranial ultrasound and intravenous microbubbles on blood brain barrier permeability in a large animal model
SCHLESINGER I, 2015, PARKINSONS DIS-US	10.1155/2015/219149	ARTICLE	PARKINSONS DIS-US	2015	SCHLESINGER, I	RAMBAM HLTH CARE CAMPUS, ISRAEL	54	7.71	0.841	Human	Movement disorder	MRI guided focused ultrasound thalamotomy for moderate-to-severe tremor in Parkinson’s disease
DIAZ RJ, 2014, NANOMED-NANOTECHNOL	10.1016/j.nano.2013.12.006	ARTICLE	NANOMED-NANOTECHNOL	2014	RUTKA, JT	UNIV TORONTO, CANADA, CANADA	54	6.75	0.829	*In vivo*	Blood-brain barrier	Focused ultrasound delivery of Raman nanoparticles across the blood-brain barrier: potential for targeting experimental brain tumors
DEFFIEUX T, 2010, IEEE T ULTRASON FERR	10.1109/TUFFC.2010.1738	ARTICLE	IEEE T ULTRASON FERR	2010	DEFFIEUX, T	COLUMBIA UNIV, USA	54	4.5	0.309	Animal and Human skulls	Blood-brain barrier	Numerical study of a simple transcranial focused ultrasound system applied to blood-brain barrier opening
LEGON W, 2018, HUM BRAIN MAPP	10.1002/hbm.23981	ARTICLE	HUM BRAIN MAPP	2018	LEGON, W	UNIV VIRGINIA, USA	53	13.25	0.809	Human	Neuromodulation	Neuromodulation with single-element transcranial focused ultrasound in human thalamus
MEAD BP, 2017, NANO LETT	10.1021/acs.nanolett.7b00616	ARTICLE	NANO LETT	2017	HANES, J; PRICE, RJ	JOHNS HOPKINS UNIV, USA; UNIV VIRGINIA, USA	52	10.4	0.933	Animal	Blood-brain barrier	Novel focused ultrasound gene therapy approach noninvasively restores dopaminergic neuron function in a rat Parkinson’s disease model
LIN CY, 2016, J CONTROL RELEASE	10.1016/j.jconrel.2016.05.052	ARTICLE	J CONTROL RELEASE	2016	LIU, HL	CHANG GUNG UNIV, TAIWAN	51	8.5	0.481	Animal	Blood-brain barrier	Non-invasive, neuron-specific gene therapy by focused ultrasound-induced blood-brain barrier opening in Parkinson’s disease mouse model
ELIAS WJ, 2013, J NEUROSURG	10.3171/2013.5.JNS122327	ARTICLE	J NEUROSURG	2013	ELIAS, WJ	UNIV VIRGINIA, USA	51	5.67	0.395	Animal	Lesion size	A magnetic resonance imaging, histological, and dose modeling comparison of focused ultrasound, radiofrequency, and Gamma Knife radiosurgery lesions in swine thalamus
SUN T, 2015, PHYS MED BIOL	10.1088/0031-9155/60/23/9079	ARTICLE	PHYS MED BIOL	2015	SUN, T	HARVARD UNIV, USA, USA	50	7.14	0.779	Animal	Blood-brain barrier	Acoustic cavitation-based monitoring of the reversibility and permeability of ultrasound-induced blood-brain barrier opening
MCDANNOLD N, 2003, MAGNET RESON MED	10.1002/mrm.10453	ARTICLE	MAGNET RESON MED	2003	MCDANNOLD, N	BRIGHAM & WOMENS HOSP, USA	48	2.53	1	Animal	Technique	MRI-guided focused ultrasound surgery in the brain: tests in a primate model
BOUTET A, RANJAN M, 2018, BRAIN	10.1093/brain/awy278	ARTICLE	BRAIN	2018	LOZANO, AM	UNIV TORONTO, CANADA, CANADA	47	11.75	0.718	Human	Movement disorder	Focused ultrasound thalamotomy location determines clinical benefits in patients with essential tremor
TIMBIE KF, 2017, J CONTROL RELEASE	10.1016/j.jconrel.2017.03.017	ARTICLE; PROCEEDINGS PAPER	J CONTROL RELEASE	2017	PRICE, RJ	UNIV VIRGINIA, USA	46	9.2	0.825	Animal	Blood-brain barrier	MR image-guided delivery of cisplatin-loaded brain-penetrating nanoparticles to invasive glioma with focused ultrasound
JUNG HH, 2015, J NEUROSURG	10.3171/2014.8.JNS132603	ARTICLE	J NEUROSURG	2015	CHANG, JW	YONSEI UNIV, SOUTH KOREA	45	6.43	0.701	Human	Movement disorder	Different magnetic resonance imaging patterns after transcranial magnetic resonance–guided focused ultrasound of the ventral intermediate nucleus of the thalamus and anterior limb of the internal capsule in patients with essential tremor or obsessive-compulsive disorder
NA YC, 2015, NEUROLOGY	10.1212/WNL.0000000000001826	EDITORIAL MATERIAL	NEUROLOGY	2015	CHANG, JW	YONSEI UNIV, SOUTH KOREA	44	6.29	0.685	Human	Movement disorder	Unilateral magnetic resonance–guided focused ultrasound pallidotomy for Parkinson’s disease
CHAUVET D, 2013, J NEUROSURG	10.3171/2013.1.JNS12559	ARTICLE	J NEUROSURG	2013	AUBRY, JF	ESPCI, INST LANGEVIN, FRANCE	43	4.78	0.333	Human	Technique	Targeting accuracy of transcranial magnetic resonance-guided high-intensity focused ultrasound brain therapy: a fresh cadaver model
HUANG YX, 2017, RADIOLOGY	10.1148/radiol.2016152154	ARTICLE	RADIOLOGY	2017	HUANG, YX	SUNNYBROOK RES INST, CANADA	42	8.4	0.754	Human	Blood-brain barrier	Opening the blood-brain barrier with MR imaging–guided focused ultrasound: preclinical testing on a trans–human skull porcine model
CHAZEN JL, 2018, J NEUROSURG	10.3171/2017.4.JNS162803	ARTICLE	J NEUROSURG	2018	CHAZEN, JL	Weill Cornell Medicine, USA	41	10.25	0.626	Human	Movement disorder	Clinical improvement associated with targeted interruption of the cerebellothalamic tract following MR-guided focused ultrasound for essential tremor
COHEN ZR, 2007, NEUROSURGERY	10.1227/01.NEU.0000245606.99946.C6	ARTICLE	NEUROSURGERY	2007	RAM, Z	TEL AVIV MED CTR & SCH MED, ISRAEL	41	2.73	0.599	Animal	Technique	Magnetic resonance imaging-guided focused ultrasound for thermal ablation in the brain: a feasibility study in a swine model
COLUCCIA D, 2018, NANOMED-NANOTECHNOL	10.1016/j.nano.2018.01.021	ARTICLE	NANOMED-NANOTECHNOL	2018	RUTKA, JT	HOSP SICK CHILDREN, CANADA	40	10	0.611	Animal	Blood-brain barrier	Enhancing glioblastoma treatment using cisplatin-gold-nanoparticle conjugates and targeted delivery with magnetic resonance-guided focused ultrasound
MONTEITH SJ, 2013, J NEUROSURG-a	10.3171/2012.12.JNS121095	ARTICLE	J NEUROSURG	2013	MONTEITH, S	UNIV VIRGINIA, USA	40	4.44	0.31	Animal	ICH	Minimally invasive treatment of intracerebral hemorrhage with magnetic resonance–guided focused ultrasound
LIPSMAN N, 2014, NEUROTHERAPEUTICS	10.1007/s13311-014-0281-2	REVIEW	NEUROTHERAPEUTICS	2014	LIPSMAN, N	UNIV TORONTO, CANADA, CANADA	39	4.88	0.599	Review		Intracranial applications of magnetic resonance-guidedfocused ultrasound
PULKKINEN A, 2014, PHYS MED BIOL	10.1088/0031-9155/59/7/1679	ARTICLE	PHYS MED BIOL	2014	PULKKINEN, A	UNIV EASTERN FINLAND, FINLAND	39	4.88	0.599	Human	Technique	Numerical simulations of clinical focused ultrasound functional neurosurgery
RAVIKUMAR VK, 2017, MOVEMENT DISORD	10.1002/mds.26997	ARTICLE	MOVEMENT DISORD	2017	HALPERN, CH	STANFORD UNIV, USA	38	7.6	0.682	Human	Movement disorder	Cost-effectiveness of focused ultrasound, radiosurgery, and DBS for essential tremor
WINTERMARK M, 2014, RADIOLOGY	10.1148/radiol.14132112	ARTICLE	RADIOLOGY	2014	WINTERMARK, M	UNIV VIRGINIA, USA	37	4.62	0.568	Human	Movement disorder	Thalamic connectivity in patients with essential tremor treated with MR imaging–guided focused ultrasound: *in vivo* fiber tracking by using diffusion-tensor MR imaging
MEDEL R, 2012, NEUROSURGERY	10.1227/NEU.0b013e3182672ac9	REVIEW	NEUROSURGERY	2012	KASSELL, NF	UNIV VIRGINIA, USA	37	3.7	0.431	Review		Magnetic resonance–guided focused ultrasound surgery: Part 2: a review of current and future applications
FAN CH, 2017, J CONTROL RELEASE	10.1016/j.jconrel.2017.07.004	REVIEW	J CONTROL RELEASE	2017	YEH, CK	NATL TSING HUA UNIV, TAIWAN	36	7.2	0.646	Review		Ultrasound targeted CNS gene delivery for Parkinson’s disease treatment
DEVARAKONDA SB, 2017, NANO LETT	10.1021/acs.nanolett.7b00272	ARTICLE	NANO LETT	2017	BANERJEE, RK	UNIV CINCINNATI, USA	36	7.2	0.646	Phantom	Technique	Assessment of gold nanoparticle-mediated-enhanced hyperthermia using MR-guided high-intensity focused ultrasound ablation procedure
ALLI S, 2018, J CONTROL RELEASE	10.1016/j.jconrel.2018.05.005	ARTICLE	J CONTROL RELEASE	2018	RUTKA, JT	HOSP SICK CHILDREN, CANADA	35	8.75	0.534	Animal	Blood-brain barrier	Brainstem blood brain barrier disruption using focused ultrasound: a demonstration of feasibility and enhanced doxorubicin delivery
O’REILLY MA, 2017, J ULTRAS MED	10.7863/ultra.16.02005	ARTICLE	J ULTRAS MED	2017	O’REILLY, MA	SUNNYBROOK RES INST, CANADA	35	7	0.628	Animal	Blood-brain barrier	Blood-brain barrier closure time after controlled ultrasound-induced opening is independent of opening volume
MOROCZ IA, 1998, J MAGN RESON IMAGING	10.1002/jmri.1880080126	ARTICLE	J MAGN RESON IMAGING	1998	JOLESZ, FA	HARVARD UNIV, USA, USA	35	1.46	1	Animal	Complications	Brain edema development after MRI-guided focused ultrasound treatment
WANG F, 2009, J ULTRAS MED	10.7863/jum.2009.28.11.1501	ARTICLE	J ULTRAS MED	2009	CHENG, Y	CHONGQING MED UNIV, PEOPLES R CHINA	34	2.62	0.288	Animal	Blood-brain barrier	Focused ultrasound microbubble destruction-mediated changes in blood-brain barrier permeability assessed by contrast-enhanced magnetic resonance imaging
FASANO A, 2017, NEUROLOGY	10.1212/WNL.0000000000004268	ARTICLE	NEUROLOGY	2017	FASANO, A	UNIV TORONTO, CANADA, CANADA	33	6.6	0.592	Human	Movement disorder	MRI-guided focused ultrasound thalamotomy in non-ET tremor syndromes
KIM M, 2017, STEREOT FUNCT NEUROS	10.1159/000478866	ARTICLE	STEREOT FUNCT NEUROS	2017	CHANG, JW	YONSEI UNIV, SOUTH KOREA	33	6.6	0.592	Human	Movement disorder	Comparative evaluation of magnetic resonance-guided focused ultrasound surgery for essential tremor
O’REILLY MA, 2017, THERANOSTICS	10.7150/thno.20621	ARTICLE	THERANOSTICS	2017	O’REILLY, MA	SUNNYBROOK RES INST, CANADA	33	6.6	0.592	Animal	Blood-brain barrier	Investigation of the safety of focused ultrasound-induced blood-brain barrier opening in a natural canine model of aging
WEINTRAUB D, 2017, MOVEMENT DISORD	10.1002/mds.26599	REVIEW	MOVEMENT DISORD	2017	ELIAS, WJ	UNIV VIRGINIA, USA	33	6.6	0.592	Review		The emerging role of transcranial magnetic resonance imaging–guided focused ultrasound in functional neurosurgery
DOBRAKOWSKI PP, 2014, INTERV NEURORADIOL	10.15274/INR-2014-10033	REVIEW	INTERV NEURORADIOL	2014	DOBRAKOWSKI, PP	MED UNIV SILESIA, POLAND	33	4.12	0.507	Review	Movement disorder	MR-guided focused ultrasound: a new generation treatment of Parkinson’s disease, essential tremor and neuropathic pain
FISHMAN PS, 2018, MOVEMENT DISORD	10.1002/mds.27401	ARTICLE	MOVEMENT DISORD	2018	FISHMAN, PS	UNIV MARYLAND SCH MED, USA	32	8	0.489	Human	Movement disorder	Neurological adverse event profile of magnetic resonance imaging–guided focused ultrasound thalamotomy for essential tremor
MONTEITH SJ, 2013, J NEUROSURG	10.3171/2012.10.JNS12186	ARTICLE	J NEUROSURG	2013	MONTEITH, S	UNIV VIRGINIA, USA	32	3.56	0.248	Human	Pain	Transcranial magnetic resonance–guided focused ultrasound surgery for trigeminal neuralgia: a cadaveric and laboratory feasibility study
MOSER D, 2012, NEUROSURG FOCUS	10.3171/2011.10.FOCUS11246	ARTICLE	NEUROSURG FOCUS	2012	MOSER, D	CTR ULTRASOUND FUNCT NEUROSURG, SWITZERLAND	32	3.2	0.373	Human	Targetting error	Measurement of targeting accuracy in focused ultrasound functional neurosurgery
JUNG NY, 2019, J NEUROSURG	10.3171/2018.2.JNS172514	ARTICLE	J NEUROSURG	2019	CHANG, JW	YONSEI UNIV, SOUTH KOREA	31	10.33	0.589	Human	Movement disorder	The efficacy and limits of magnetic resonance–guided focused ultrasound pallidotomy for Parkinson’s disease: a Phase I clinical trial
WANG F, 2012, PLOS ONE	10.1371/journal.pone.0052925	ARTICLE	PLOS ONE	2012	CHEN, Y	PEKING UNIV, PEOPLES R CHINA	31	3.1	0.361	Animal	Blood-brain barrier	Targeted delivery of GDNF through the blood–brain barrier by MRI-guided focused ultrasound
PARK YS, 2019, MOVEMENT DISORD	10.1002/mds.27637	ARTICLE	MOVEMENT DISORD	2019	CHANG, JW	YONSEI UNIV, SOUTH KOREA	30	10	0.57	Human	Movement disorder	Four-year follow-up results of magnetic resonance-guided focused ultrasound thalamotomy for essential tremor
APPELBOOM G, 2016, NEURO-ONCOLOGY	10.1093/neuonc/now137	REVIEW	NEURO-ONCOLOGY	2016	APPELBOOM, G	STANFORD MED CTR, USA	30	5	0.283	Review		Stereotactic modulation of blood-brain barrier permeability to enhance drug delivery
KRISHNA V, 2019, NEUROSURGERY	10.1093/neuros/nyy020	ARTICLE	NEUROSURGERY	2019	KRISHNA, V	OHIO STATE UNIV, USA	29	9.67	0.551	Human	Movement disorder	Prospective tractography-based targeting for improved safety of focused ultrasound thalamotomy
MENG Y, 2019, ANN NEUROL	10.1002/ana.25604	ARTICLE	ANN NEUROL	2019	LIPSMAN, N	SUNNYBROOK RES INST, CANADA	28	9.33	0.532	Human	Blood-brain barrier	Glymphatics visualization after focused ultrasound-induced blood–brain barrier opening in humans

These articles can be divided into three topics depending on the pathology type in which MRgFUS use has been described in the article. Equal numbers of articles involved human studies and animal studies (*n* = 41). Thirty-six studies were related to blood brain barrier (BBB) disruption and only three studies of these were human studies. Twenty-five studies focused on the role of FUS in patients with movement disorders and all were human studies. Three articles discussed the role of FUS in patients with pain, and a similar number of articles discussed the role of FUS for oncological indications in humans. Eleven articles discussed the technical aspects, while one clinical article described the use of FUS in patients with obsessive-compulsive disorders (OCD).

The article that received the maximum number of citations was “Non-invasive MR Imaging–guided Focal Opening of the Blood-Brain Barrier in Rabbits” published in 2001 in *Radiology* by Hynynen et al. and cited 855 times (Hynynen et al., 2001). The next two articles in the top 100 list, published in the *New England Journal of Medicine*, described the clinical application of MRgFUS in essential tremors. The first of these, titled “A Pilot Study of Focused Ultrasound Thalamotomy for Essential Tremor,” was a pilot trial that established the safety and efficacy of focused ultrasound thalamotomy in 15 patients suffering from essential tremor (Elias et al., 2013). Another article published in 2016, titled “A Randomized Trial of Focused Ultrasound Thalamotomy for Essential Tremor,” was a multicentre trial that confirmed the efficacy of focused ultrasound thalamotomy (Elias et al., 2016). The total citations per year for this article were 62.67, the maximum out of 100 cited articles.

### Main information

**FIGURE 1:**
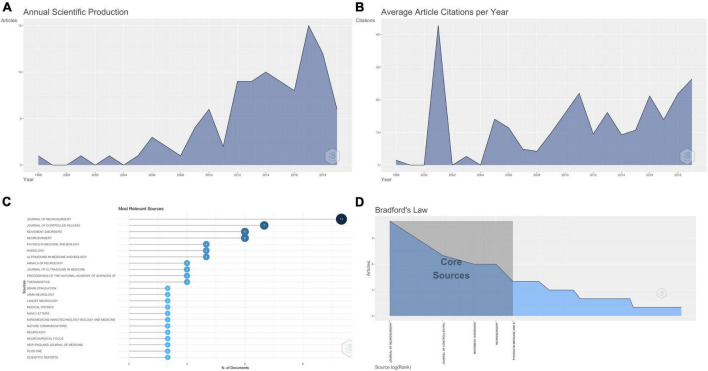
**(A)** Line graph showing the year wise number of articles published. **(B)** Line graph showing the year wise average citations per year. **(C)** Graph showing the number of articles among the top 100 cited articles on MRgFUS published in the different journals. **(D)** Graphical representation of Bradford’s law.

These 100 articles were published in 45 journals over 22 years (1998–2019). There were 85 original and 15 review articles. The average number of years from the date of publication was 7.22. Interestingly, most of these articles were published recently: 2017 (15 articles), 2018 (12 articles), and 2014 (10 articles) (Figure 1A).

Average citations per document were 97.78, while average citations per year per document were 12.47 (Table 2). Maximum mean citations per article and mean citations per year were for the articles published in 2001 and were 855 and 42.75, respectively (Figure 1B).

**TABLE 2:** Main information about data.

Description	Results
Timespan	1998:2019
Sources (Journals, Books, etc.)	45
Documents	100
Average years from publication	7.22
Average citations per documents	97.78
Average citations per year per doc	12.47
References	2,798
**Document types**	
Article	85
Article; proceedings paper	1
Editorial material	1
Review	13
**Document contents**	
Keywords Plus (ID)	365
Author’s keywords (DE)	169
**Authors**	
Authors	481
Author appearances	833
Authors of single-authored documents	0
Authors of multi-authored documents	481
**Authors collaboration**	
Single-authored documents	0
Documents per Author	0.208
Authors per document	4.81
Co-authors per documents	8.33
Collaboration index	4.81

### Journals

Figure 1C shows the top 23 journals that published two or more articles. *Journal of Neurosurgery* had a maximum of 11 publications, followed by the *Journal of Controlled Release* (7), *Movement Disorders* (6 articles), and *Neurosurgery* (6 articles). Other journals that published at least one of these articles included *Stereotactic and Functional Neurosurgery*, the *Journal of Neurology, Neurosurgery and Psychiatry*, and the *Journal of Magnetic Resonance Imaging.*

Figure 1D shows the graphical representation of Bradford’s Law, which estimates the exponentially diminishing returns of searching for references in science journals. It shows that the *Journal of Neurosurgery, Journal of Controlled Release, Movement Disorders, Neurosurgery and Physics in Medicine and Biology* lie in Zone 1.

**FIGURE 2:**
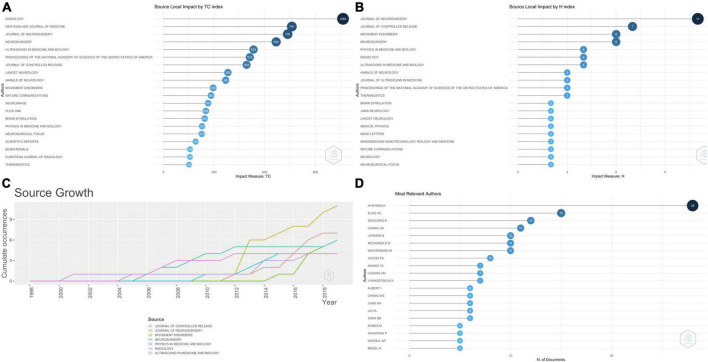
**(A)** Graph showing the top 20 journals with maximum impact in terms of total citations received by the articles included in the analysis. **(B)** Graph showing the top 20 journals with maximum impact in terms of H-index of the articles included in the analysis. **(C)** Line graph showing the “Source growth,” i.e., the cumulative number of articles published in the top seven journals. **(D)** Graph showing the number of articles among the top 100 cited articles on MRgFUS published by different authors.

Figure 2A shows the journal-wise distribution of the total citations received by these articles. The articles published in *Radiology* were cited the most (1,064 citations), followed by *New England Journal of Medicine* (761 citations) and *Journal of Neurosurgery* (736 citations). The articles published in these journals were 4, 2, and 11, respectively. Figure 2B shows the journal impact measured in the H-index, with *the Journal of Neurosurgery* at the top. Figure 2C shows the source dynamics, i.e., the year-wise increase in the number of articles. *Journal of Neurosurgery* published the first article in 2012, and there has been a rapid increase in the published articles since.

### Authors

These top 100 cited articles included 481 authors who made 833 appearances in these 100 articles. The number of co-authors per document was 8.33, with a collaboration index of 4.81. None of these articles were published by a single author.

**FIGURE 3:**
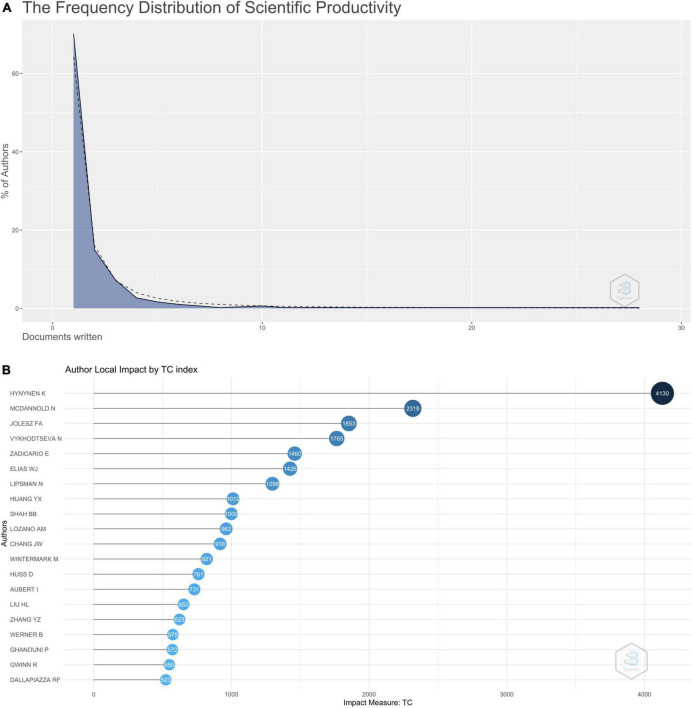
**(A)** Graphical representation of Lotka’s law. **(B)** Graph showing the top 20 authors with maximum impact as measured by total citations received by the top 100 cited articles on MRgFUS.

Figure 2D shows the top 20 authors who published the maximum number of these articles. Hynynen K authored 28 out of these top 100, followed by Elias WJ (15 articles) and Zadicario (12 articles). Supplementary Figure 1 shows the top 20 authors’ production over time, with the size of the dots denoting the number of articles and the shade of the dots denoting the number of citations per year. Figure 3A shows the graphical representation of Lotka’s law, which denotes the distribution of the number of articles published by the number of authors. Most of the authors (85%) published 1 (70%) or 2 (15%) articles. Figure 3B shows the authors whose articles received the maximum number of citations. Articles authored by Hynynen received the maximum number of citations (4130). Table 3 shows the various indexes of the top 50 authors. H-index and g-index were maximum for Hynynen K, while the m-index, which considers the h-index and the number of years an author has been active for, was maximum for Chang JW (1.5710).

**TABLE 3:** Various indexes of the top 50 authors.

Element	H Index	G Index	M Index	Total citations	Number of articles	Production year start
HYNYNEN K	28	28	1.167	4,130	28	1998
ELIAS WJ	15	15	1.5	1,426	15	2012
ZADICARIO E	12	12	0.75	1,460	12	2006
CHANG JW	11	11	1.571	918	11	2015
LIPSMAN N	10	10	1.111	1,298	10	2013
MCDANNOLD N	10	10	0.476	2,318	10	2001
WINTERMARK M	10	10	1	821	10	2012
JOLESZ FA	8	8	0.333	1,853	8	1998
HUANG YX	7	7	0.583	1,012	7	2010
LOZANO AM	7	7	0.778	962	7	2013
VYKHODTSEVA N	7	7	0.333	1,765	7	2001
AUBERT I	6	6	0.5	731	6	2010
CHANG WS	6	6	0.857	394	6	2015
JUNG HH	6	6	0.857	394	6	2015
LIU HL	6	6	0.5	653	6	2010
SHAH BB	6	6	0.667	1,000	6	2013
EAMES M	5	5	0.5	239	5	2012
GHANOUNI P	5	5	0.714	572	5	2015
KASSELL NF	5	5	0.385	252	5	2009
MEDEL R	5	5	0.385	252	5	2009
O’REILLY MA	5	5	0.625	247	5	2014
SCHWARTZ ML	5	5	0.556	493	5	2013
SNELL J	5	5	0.5	239	5	2012
YEH CK	5	5	0.556	371	5	2013
AUBRY JF	4	4	0.333	238	4	2010
FAN CH	4	4	0.444	320	4	2013
GWINN R	4	4	0.667	550	4	2016
HANES J	4	4	0.5	310	4	2014
HUSS DS	4	4	0.5	247	4	2014
KONOFAGOU EE	4	4	0.333	231	4	2010
MENG Y	4	4	1	439	4	2018
MONTEITH SJ	4	4	0.4	494	4	2012
PRICE RJ	4	4	0.5	310	4	2014
SHEEHAN JP	4	4	0.308	196	4	2009
WANG F	4	4	0.308	199	4	2009
WERNER B	4	4	0.308	575	4	2009
ZHANG YZ	4	4	0.364	623	4	2011
BLACK SE	3	3	0.75	310	3	2018
BOCH AL	3	3	0.25	187	3	2010
CHENG Y	3	3	0.231	168	3	2009
DALLAPIAZZA RF	3	3	0.429	523	3	2015
EISENBERG HM	3	3	0.5	463	3	2016
FINK M	3	3	0.25	187	3	2010
FISHMAN PS	3	3	0.5	463	3	2016
HARNOF S	3	3	0.188	206	3	2006
HEYN C	3	3	0.75	411	3	2018
JEANMONOD D	3	3	0.231	513	3	2009
JUNG NY	3	3	0.6	94	3	2017
KLIBANOV AL	3	3	0.375	264	3	2014
KRISHNA V	3	3	0.75	124	3	2018

### Affiliations and country

**FIGURE 4:**
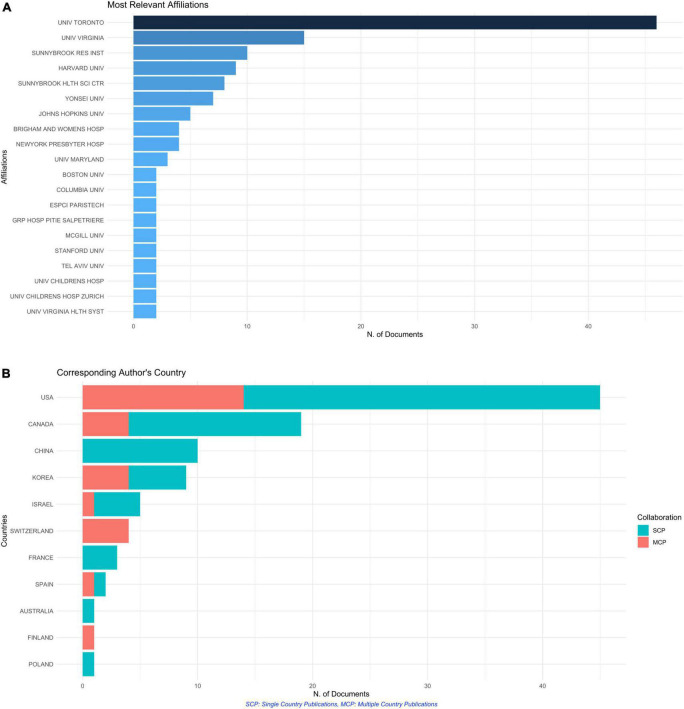
**(A)** Bar graph showing the top 20 affiliations of the authors who authored these top 100 cited articles. **(B)** Bar graph showing the countries which published the top 100 cited articles. Orange indicates publications with authors from more than one country (i.e., multicountry collaboration).

Figure 4A shows the top 20 universities which published the maximum number of articles. The maximum number of authors belong to the University of Toronto. Most universities were from the United States, Canada, Israel, and Taiwan.

**FIGURE 5:**
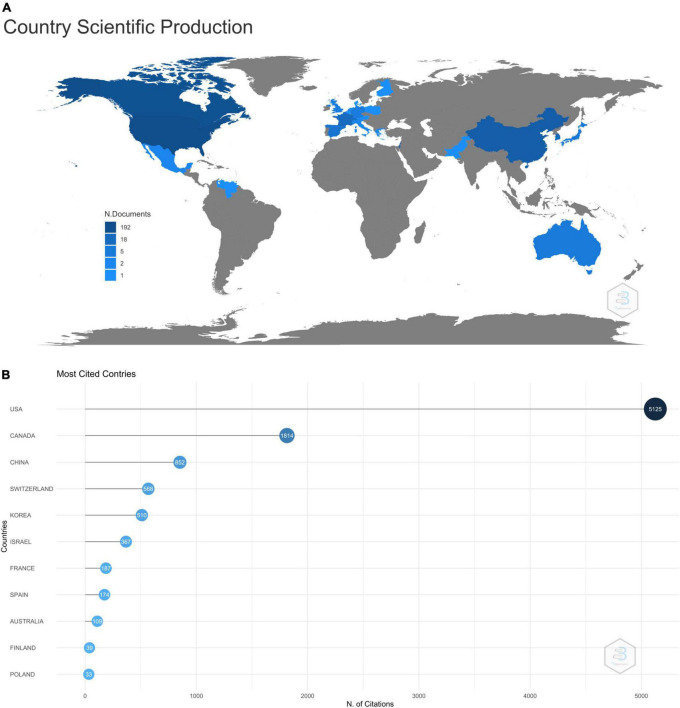
**(A)** Shade graded world map showing the distribution of scientific publications per the total number of authors from a country with the darker shade implying a greater number of authors from a country. **(B)** Graph showing the number of citations received by the articles published from various countries (as per the country of the corresponding author).

These top 100 cited articles were authored by corresponding authors from 11 countries, showing the limited availability of this technology. Figure 4B shows the country-wise distribution of the corresponding authors of these top 100 cited articles, with the maximum number of articles (*n* = 45) published from the United States. It was followed by Canada (19 articles) and China (10 articles). There were significant international collaborations in these articles, as shown by the orange bars in Figure 4B. The MCP ratio (Multicountry production ratio, which is the ratio of articles authored by authors belonging to more than one country to those authored by authors from one single country) was maximum (= 1) for the articles published by the authors from Switzerland and Finland, suggesting that all their articles had multicountry collaborations. Even the articles published from the United States had an MCP ratio of 0.31, with 14 out of 45 articles having authors from countries other than the United States. Figure 5A shows the world map with different shades of blue, showing the countries of all the authors (not corresponding authors alone) who were part of the author list of these 100 articles. It shows that most of the authors belonged to North America and Europe. Figure 5B shows the number of times the articles from a given country (as per the corresponding author) were cited. The articles from the United States were cited 5,125 times, followed by Canada (1,814 citations) and China (852 citations). However, average citations per article were maximum for the articles published from Switzerland (142), followed by the articles from the United States (113.9).

**FIGURE 6:**
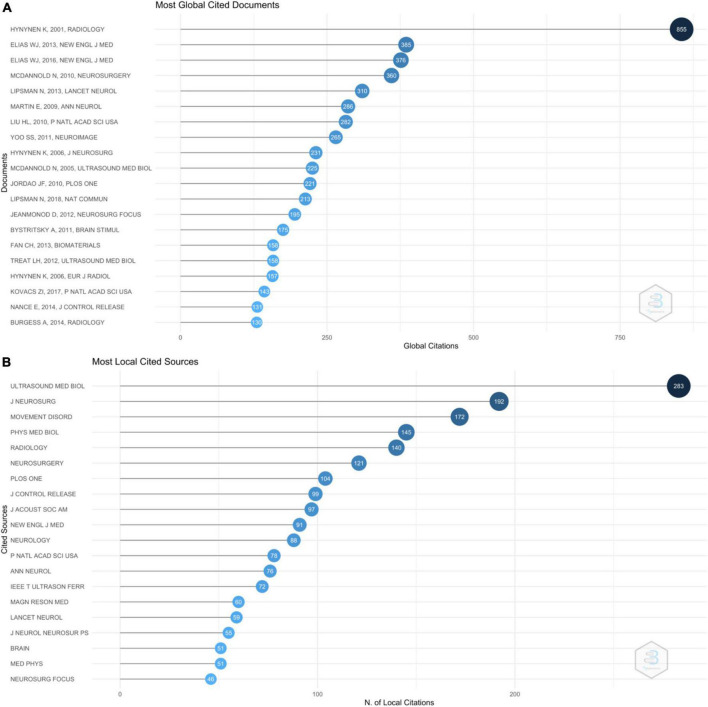
**(A)** Graph showing the number of times the top 20 cited articles were cited in all the journals. **(B)** Graph showing the number of times these top 100 cited articles were cited in these top 100 cited articles.

Figure 6A shows the number of times the top 20 cited articles were cited in all the journals, with the article by Hynynen et al. published in Radiology in 2011 cited 855 times. Figure 6B shows the number of times these top 100 cited articles were cited in these top 100 cited articles. Article by Hynynen et al. published in Radiology in 2011 was cited in 40 out of these top 100 cited articles.

Supplementary Figure 2 shows the most commonly used keywords in these top 100 cited articles. The most common keyword was “focused ultrasound,” followed by “blood-brain barrier” and “essential tremor.”

Supplementary Figure 3 shows the three-field plot between the author country (left field), author (center field), and the keywords (right field). It can be appreciated that the countries with a maximum number of connections were Canada and the United States of America (USA). In contrast, the authors with the maximum number of connections were Hynynen K, Lozano AM, Lipsman N, and Elias WJ.

## Discussion

MRgFUS is one of the most evolving field in the neurosurgery This scientometric analysis of the MRgFUS revealed some interesting and intriguing trends. First, these articles were published over the last 22 years, from 1998 to 2019. Obviously, it takes a few years for any article to garner many citations to feature in the top 100 cited articles and more importantly adoption in the clinical field given novelty of the technology. However, articles published recently, as recent as in 2019, appeared in this top 100 list. This reflects the rapid evolution and the acceptance of the field, not only by clinician but also the patients. As this technology is evolving and explored in newer neuropsychiatric indications, the scientific publications and their citations are going to be more voluminous and robust in near future.

Secondly, only 11 countries contributed to these top 100 cited articles, with the top 2 countries (the United States and Canada) contributing to more than half of these articles. As MRgFUS is a new technology and its availability is yet limited. There was no article from low to middle-income countries, perhaps due to the high cost of initial set up. The low acceptance of a surgical procedure for non-life-threatening diseases may also contribute to the limited interest of many countries. Although the average citations per document of these articles were less when compared with the top 100 cited articles on other pathologies, the average citations per document per year were higher (Agrawal et al., 2021). This reflects not only that most of these articles were published recently but also that they are being increasingly cited and applied increasingly to clinical and translational research.

The analysis revealed that 41 articles were based on animal or cadaveric studies to study the preclinical aspects of focused ultrasound. It again reflects the novelty of the technology and value of translational impact in clinical neurosurgery. Amongst the human studies, most of the articles (*n* = 25) described the use of MRgFUS in various movement disorders, with essential tremor being the commonest pathology. The other rare pathologies where the use of MRgFUS has been described in these studies are Parkinson’s disease, dystonia, and OCD.

There was one animal study and four human trials in the top five cited articles (Hynynen et al., 2001; McDannold et al., 2010; Elias et al., 2013, 2016; Lipsman et al., 2014). The highest cited article was an animal study. Hynynen et al. studied if focused ultrasound beams can be used to locally open the blood-brain barrier without damage to surrounding brain tissue and if magnetic resonance (MR) imaging can be used to monitor this procedure in this article (Hynynen et al., 2001). The authors established that the BBB could be consistently opened with transcranial FUS, opening a new translational field in neurological disorders.

Three out of four human trials were proof of concept studies or pilot studies (McDannold et al., 2010; Elias et al., 2013, 2016). The second most cited article was a pilot study by Elias et al. and published in *NEJM* in 2013 (Elias et al., 2013). The authors reported total tremor and disability scores improved from 54.9 to 24.3 (*P* = 0.001) and 18.2 to 2.8 (*P* = 0.001), respectively in 15 ET patients treated with FUS thalamotomy. This pilot trial was followed by a multicentric randomized controlled trial, published in *NEJM* in 2016, comparing the efficacy of unilateral focused ultrasound thalamotomy with a sham procedure in 76 patients with ET (Elias et al., 2016). The authors observed that hand-tremor scores improved more after focused ultrasound thalamotomy than after sham procedure, and the improvement was maintained 12 months after thalamotomy. Improvement in secondary outcome measures assessing disability and quality of life was also noticed.

These 100 top cited articles were published in in 45 various neurosurgical, neurological, radiological, and basic sciences research journals, including high-impact journals like *NEJM, JAMA Neurology, Nature communications, Annals of Neurology, Movement Disorders, Radiology, and Neurosurgery*. This diversity may represent the interest from different specialties, leading to faster advancements in this technology and a steep increase in the amount of literature. *Journal of Neurosurgery* published 11 out of these top 100, perhaps related to the fact that neurosurgeons are the end users of this technology and have been actively involved in FUS-related research.

The top-cited article by Hynynen guided *Radiology* to be the maximally cited journal out of all the journals in which these articles were published (Hynynen et al., 2001).

One of the important finding noted in this study was that the number of co-authors per document was 8.33, indicating that these articles were published by research teams with larger collaborative members and/or multicentric collaboration. Moreover, 15% of the authors contributed to more than two articles in the top 100 cited articles. Hynynen K, a Professor of Medical Biophysics at the University of Toronto, was the top contributor in these journals, contributing to 28 out of these top 100 cited articles, followed by Elias WJ (15 articles) and Zadicario (12 articles). The second highest contributor to these articles is Elias WJ, a Professor of Neurological Surgery at the University of Virginia. The third highest contributor was Eyal Zadicario, who is a part of the InSightec (manufacturer) team. Hynynen K has contributed to FUS research for more than three decades and is the lead author of the highly cited article in this list.

### Limitations

There are some inherent drawbacks of bibliometric analysis. The reasons why a paper is cited multiple times may be diverse and may not accurately reflect the influence of the study in question (Garfield, 1979). Sole reliance on these indicators can lead to missing specific papers reporting (Allen et al., 2009). Similarly, recent publications on the topic and young researchers might not have accrued enough citations to make it to the list of top 100 articles. One of the inherent limitation of this study is that the search criteria included both the animal and human studies. It is not uncommon to have lesser citation of the animal studies than human studies, given recent and increasingly more acceptance in clinical trials and clinical practise. The citation matrix alone should not be taken as the sole criteria about the value and rigor of the study. The search was conducted on Web of Science, the most common database used for bibliometric analyses, but still, fallacious exclusions of some articles could have happened due to the keyword-specific results obtained.

## Conclusion

MRgFUS is one of the fastest evolving field in neurosurgery, specifically functional neurosurgery with increasing studies in recent years. As novel indications are studied, it is crucial to identify the most important topics and contributors to the field as scientific literature expands to guide clinician and research in the field. Most of the top 100 cited articles comes from North America and Europe, with the United States and Canada contributing to more than half of the articles on MRgFUS. The top 100 cited articles also highlight the access of MRgFUS to developed countries and healthcare disparity in access of MrgFUS to developing countries.

## Author contributions

KG: conceptualization, methodology, software, writing, rewriting and editing, data curation, writing—original draft preparation, visualization, investigation, software, and validation. MR: conceptualization, writing, rewriting and editing, writing—original draft preparation, visualization, investigation, supervision, software, and validation. VK: conceptualization, methodology, rewriting and editing, and supervision. MS and AR: methodology, rewriting and editing, and supervision. All authors contributed to the article and approved the submitted version.

## Supplementary material

Supplementary Figure 1 Graph showing the year wise distribution of number of articles among the top 100 cited articles on MRgFUS published by different authors and the number of citations received by articles published in different years.

Supplementary Figure 2 Graph showing most frequently used keywords in the top 100 cited articles on MRgFUS.

Supplementary Figure 3 Three fields plot showing the predominant countries, surgeons and the key areas of work from the top 100 cited articles on MRgFUS.
