# Supplementary figures and images for: A scientometric analysis of the 100 most cited articles on magnetic resonance guided focused ultrasound
Source: Front Hum Neurosci. 2022 Sep 12;16:981571. doi: 10.3389/fnhum.2022.981571 (PMC9511032; doi:10.3389/fnhum.2022.981571)

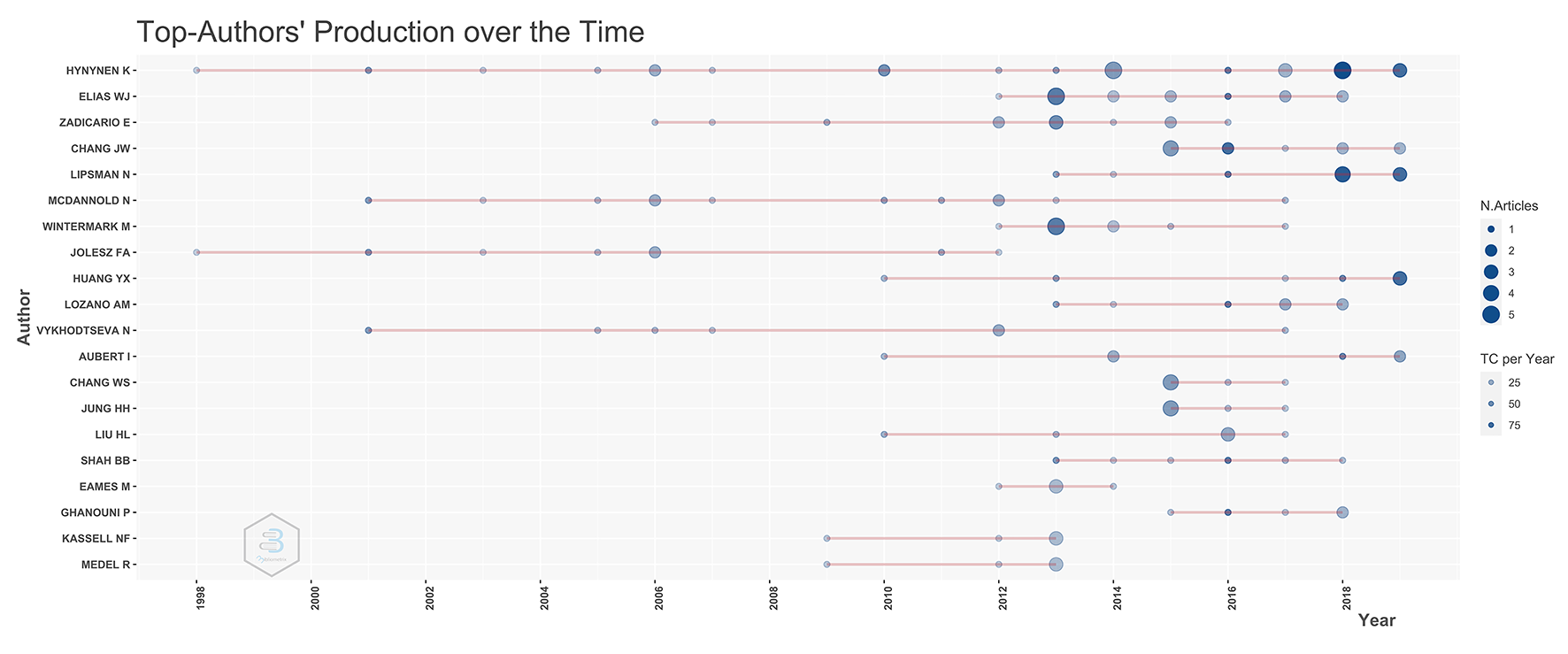

Supplement: Supplementary Figure 1 — Graph showing the year wise distribution of number of articles among the top 100 cited articles on MRgFUS published by different authors and the number of citations received by articles published in different years. [file Image_1.TIFF]

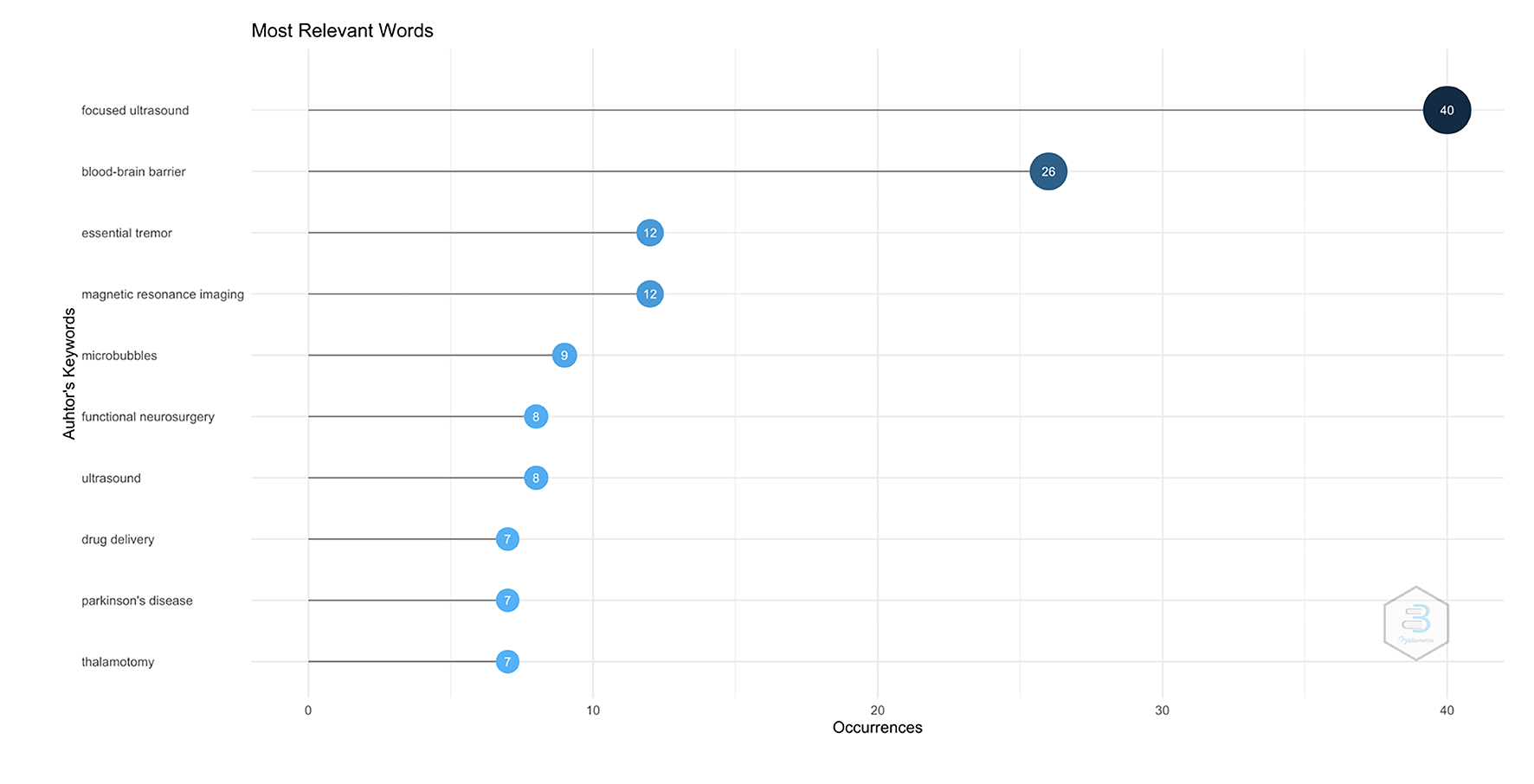

Supplement: Supplementary Figure 2 — Graph showing most frequently used keywords in the top 100 cited articles on MRgFUS. [file Image_2.TIFF]

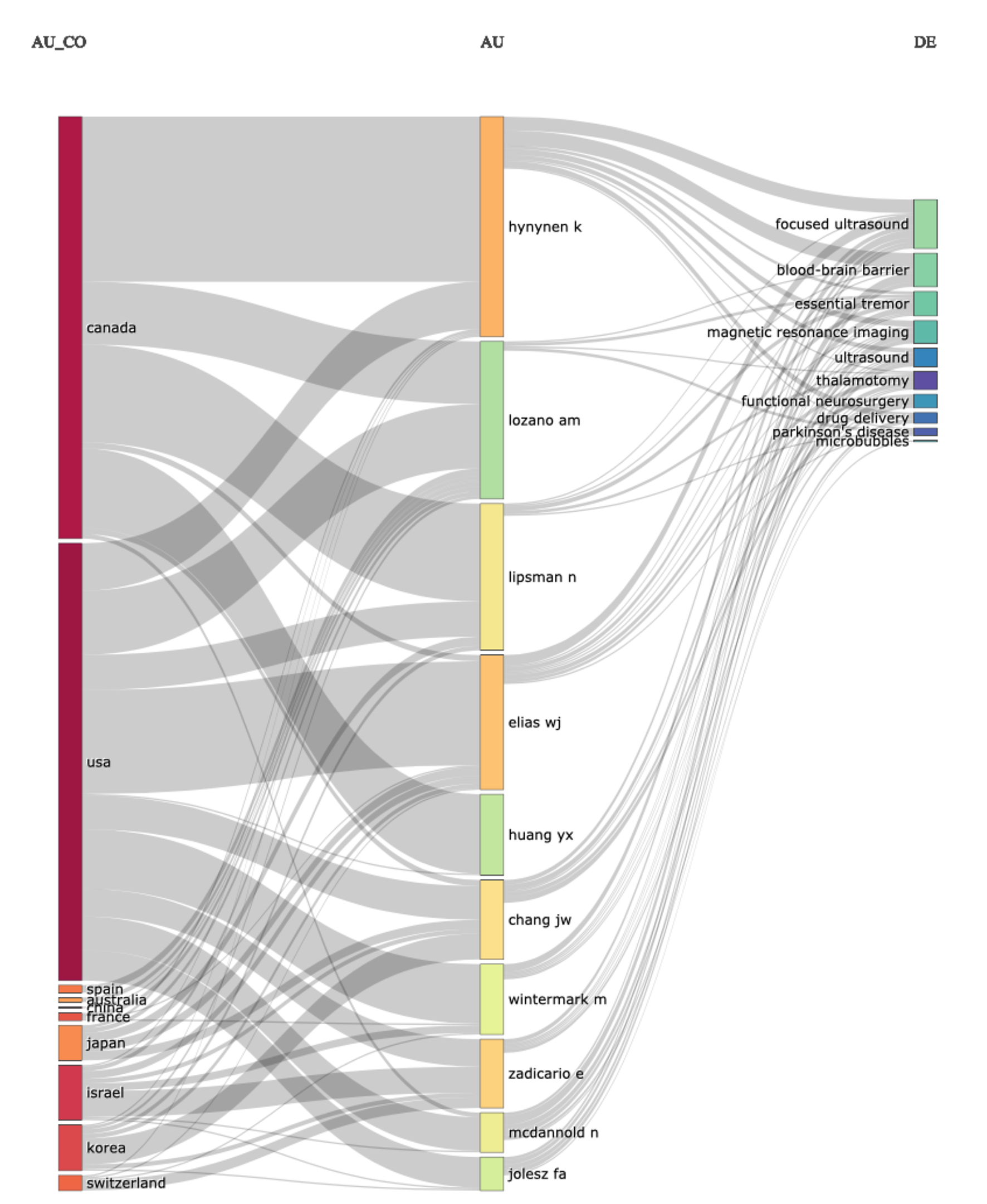

Supplement: Supplementary Figure 3 — Three fields plot showing the predominant countries, surgeons and the key areas of work from the top 100 cited articles on MRgFUS. [file Image_3.TIFF]
